# Proteomic pattern of breast milk discriminates obese mothers with infants of delayed weight gain from normal‐weight mothers with infants of normal weight gain

**DOI:** 10.1002/2211-5463.12610

**Published:** 2019-03-15

**Authors:** Christo Atanassov, Etienne Viallemonteil, Charlotte Lucas, Marylise Perivier, Stéphane Claverol, Roland Raimond, Régis Hankard

**Affiliations:** ^1^ CHU ‐ La Milétrie Poitiers France; ^2^ UMR‐CNRS 7267 Université de Potiers France; ^3^ Pédiatrie Multidisciplinaire‐Nutrition de l'Enfant CHU ‐ La Milétrie Poitiers France; ^4^ INSERM CIC 1402 Poitiers France; ^5^ Pôle Protéomique Université Victor Segalen ‐ Bordeaux 2 France; ^6^ INSERM U1069 Université François Rabelais Tours France

**Keywords:** breast milk, infant weight gain, maternal obesity, pIgR, SELDI biomarker

## Abstract

We previously reported that exclusively breastfed infants born to mothers with pregestational obesity gain less weight during the first month after birth than those born to mothers of normal pregestational weight. This issue is potentially important since lower weight gain in breastfed infants of obese mothers might increase the risk of developing later obesity. Breast milk quality and quantity, together with breastfeeding practice, possibly influence infants’ feeding behavior, appetite control, and regulation of growth later in life. The issue of whether breast milk protein patterns from obese mothers differ in composition from those of non‐obese mothers remains largely unexplored. Here, we established a breast milk proteomic pattern that discriminates obese mothers and infants with delayed weight gain at 1 month after birth from normal‐weight mothers with infants of the same age and with normal weight gain. Obese mothers were matched to normal‐weight mothers (*n* = 26; body mass index 33.5 ± 3.2 *vs* 21.5 ± 1.5 kg·m^−2^). The mean weight gain of infants in the obese group at 1 month after birth was 430.8 g lower than that of the infants in the control group. Analysis of the breast milk delipidized fraction by surface‐enhanced laser desorption/ionization on CM10 and Q10 arrays was followed by MS‐assisted purification and LC‐MS/MS microsequencing of a selected biomarker. We identified 15 candidate protein biomarkers, seven of which were overexpressed in the obese group and eight in the normal‐weight group. One of the most significant candidate biomarkers, overexpressed in the obese group, was identified as a fragment of the sixth extracellular domain of the polymeric immunoglobulin receptor. Further structural identification of these candidate biomarkers and their validation in clinical assays may facilitate the development of a predictive immunoassay.

**Abbreviations**
AUC: area under the ROC curve
BMI: body mass index
EDM: expression difference mapping
PCA: principal component analysis
pIgR: polymeric immunoglobulin receptor
ROC: receiver operating characteristic
SELDI: surface‐enhanced laser desorption/ionization

We have previously reported that exclusively breastfed infants born to mothers with pregestational obesity gain less weight during the first month after birth than those of mothers of normal pregestational weight 1. This issue is potentially important since the lower weight gain in breastfed infants of obese mothers might increase the risk of developing later obesity 2, 3 through mechanisms of catch‐up growth, such as observed in 5‐year‐old children whose body weight at birth is below normal 4. Although the perception of inadequate milk supply by obese mothers was correlated to poor weight gain of their infants 1, and growth velocity in the first months of postnatal life was associated with later overweight and obesity 5, the issue is much more complex. Breast milk quality and quantity together with breastfeeding practice possibly influence infants feeding behavior, appetite control and regulation of growth later in life. A multitude of bioactive breast milk components must be taken into consideration such as oligosaccharides, polyunsaturated fatty acids, proteins and derived peptides 6, 7, 8, cytokines 9, and hormones 10. For example, the presence of the anorexigenic hormone leptin in breast milk of non‐obese mothers at 1 month of lactation was found to be negatively correlated with infants’ body mass index (BMI) at 18 and 24 months of age 11. In this regard, we have recently reported that human milk leptin concentration was almost twice as high in obese mothers than in normal‐weight mothers at 1 month postpartum 12. Along with leptin, other yet unidentified milk components may be involved in this phenomenon.

The issue of whether breast milk protein patterns from obese mothers differ in composition from those of non‐obese mothers remains largely unexplored. To that end, we compared breast milk protein patterns in fully breastfeeding women, either obese or normal weight, by surface‐enhanced laser desorption/ionization (SELDI) ProteinChip technology. The latter allows rapid detection and semi‐quantification of candidate protein biomarkers and facilitates their further purification in view of sequencing by MS.

## Subjects, materials and methods

### Participants and clinical study design

Mother and infant dyads were recruited in the university maternity hospitals of Poitiers and Châtellerault in western France. Obese mothers (BMI ≥ 30 kg·m^−2^ before pregnancy) were matched for age (± 5 years), pregnancy status (first infant *vs* second or more), ethnic origin, and educational level with normal‐weight mothers (18.5 ≤ BMI <25 kg·m^−2^). Pairs of mothers with preexisting chronic or gestational diseases, smokers during pregnancy, twin pregnancies, children born prematurely, i.e. with a gestational age < 37 weeks of amenorrhea, with a low birth weight (< 3rd percentile for gestational age according to reference curves from the French AUDIPOG study) 13 or hospitalized in the neonatal period were excluded. Only infants who remained exclusively breastfed at 1 month and their mothers participated in this study and the study was conducted according to the principles expressed in the Declaration of Helsinki. The CPP Ouest III ethics committee approved the protocol (7 September 2009). After having explained the study protocol to all participating mothers, a written consent was obtained. Participating mothers at 1 month postpartum and their infants were investigated in the department of INSERM ‘CIC 1402’ located in the university hospital center of Poitiers, CHU – La Milétrie. Clinical examination and collection of the data presented in Data S1 were carried out as described previously 12.

### Breast milk

Milk was collected from the breast opposite to that suckled by the baby using an electric breast pump (‘Symphony’, Medela, Switzerland), immediately homogenized by vortexing, supplemented with a mix of protease inhibitors (Complete, EDTA‐free; Roche Diagnostics, Mannheim, Germany) according to the provider's protocol and stored at −80 °C. The samples were blind‐coded, with the same identification number for each pair. After a low‐speed centrifugation (1000 ***g***, 20 min, 4 °C), the intermediate layer between the lipids on the surface and the cell debris pellet was aspirated and centrifuged again (18 000 ***g***, 90 min, 4 °C). The total protein content of the aspirated intermediate layer (delipidized fraction) was determined by bicinchoninic acid assay (Pierce Biotechnology, Rockford, IL, USA).

### ProteinChip array processing

Two types of SELDI ProteinChip ion‐exchange arrays, Q10 and CM10, were assembled in a 96‐well bioprocessor (Bio‐Rad, Hercules, CA, USA) and preactivated for 30 min with their respective buffers (100 mm Tris/HCl, pH 9.0 or 100 mm sodium acetate, pH 4.0). In the next step, 180 μL of the binding buffer for the respective array was mixed with 20 μL of prediluted delipidized milk fraction (previously adjusted to a final protein concentration of 0.5 mg·mL^−1^ in all test samples) and further incubated for 60 min. All samples were tested in duplicate. After two washes with the binding buffers and one quick rinse with HPLC‐grade water, the spots were loaded twice with 1 μL of saturated solution of sinapinic acid dissolved in 50% acetonitrile/0.5% trifluoroacetic acid (v/v). All steps were carried out at room temperature (18–20 °C), using a Micromix‐5 platform shaker and a Biomek 3000 robot‐pipetting workstation (Beckman‐Coulter, Brea, CA, USA). The arrays were processed in a PCS 4000 ProteinChip Reader (Bio‐Rad), which was programmed in a positive ion mode and at ion acceleration potential of 20 kV.

For spectra processing, expression difference mapping (EDM) was carried out using proteinchip data manager 3.0.7 software (Bio‐Rad). After calibration and normalization of all spectra using the total ion current method, clusters of peaks with the same mass were defined at the following settings: signal‐to‐noise ratio (S/N; first pass), ≥ 5; minimum peak threshold, 20%; mass error, 0.3%; S/N (second pass), ≥ 2.

### Statistics

Heat map hierarchical clustering, non‐parametric Mann–Whitney test and receiver operating characteristic (ROC) curve analysis were applied for data analysis using the build‐in proteinchip data manager 3.0.7 package. In addition, paired *t*‐test, ROC curve (prism version 5.01; GraphPad Software Inc., La Jolla, CA, USA) and principal component analysis (PCA; r‐3.2.2; https://CRAN.R-project.org/doc/FAQ/R-FAQ.html; K. Hornik, 2016) were applied.

### Protein purification procedures

Ion‐exchange chromatography, reversed phase HPLC, tricine SDS/PAGE and MS‐assisted control of protein purity were conducted as described previously 14.

### Mass spectrometry analysis

Protein‐containing bands were excised from the SDS/PAGE gel and treated with trypsin overnight at 37 °C. The peptide mixture was analyzed on a Dionex U‐3000 Ultimate nano‐LC system coupled to a nanospray LTQ‐Orbitrap XL mass spectrometer (Thermo‐Finnigan, San Jose, CA, USA). Data were acquired in a data‐dependent mode alternating an MS scan survey over the range *m*/*z* 300–1700 and six MS/MS scans in an exclusion dynamic mode. MS/MS spectra were acquired using a 3 *m*/*z* units ion isolation window, a 35% relative collision energy, and a 30 s dynamic exclusion duration. Data were searched by sequest through a proteome discoverer 1.3 interface (Thermo‐Finnigan) against a subset of the 2012.09 version of UniProt database restricted to *Homo sapiens* (67 949 entries). The search parameters were as follows: mass accuracy of the monoisotopic peptide precursor and peptide fragments was set to 10 p.p.m. and 0.6 Da, respectively. Only *b*‐ and *y*‐ions were considered for mass calculation. Oxidation of methionines (+16 Da) was considered as variable modification. Two missed trypsin cleavages were allowed. Peptide validation was performed and only ‘high confidence’ peptides were retained corresponding to a 1% false positive rate at peptide level.

## Results

The present study relies on a careful selection of the studied subjects in the two groups by the following principal characteristics: close age and large BMI difference of the mothers; close values of the infants’ weight at birth and great delay in the infants’ weight gain after 1 month of breastfeeding. The difference between the means of infants’ weight gain of the normal‐weight and the obese groups was 430.8 ± 97.1 g (952.5 ± 71.3 g in the normal‐weight group *vs* 521.7 ± 65.9 g in the obese group). This weight gain in favor of infants of normal‐weight mothers was significant when analyzed by paired *t*‐test (*P* < 0.0001), and the area under the ROC curve (AUC) was 0.82 (Table 1).

**Table 1:** Principal characteristics of the mother–infant dyads

	Normal‐weight dyads (*n* = 26)	Obese dyads (*n* = 26)
Mothers		
Age (years)	30.7 ± 4.5	31.4 ± 4.8
BMI (kg·m^−2^)	21.5 ± 1.5	33.5 ± 3.2
Infants’ weight at birth (g)	3 462.5 ± 387.2	3 522.3 ± 566.7
Infants’ weight at 1 month (g)	4 415.0 ± 465.4	4 044.0 ± 676.9
Infants’ weight gain at 1 month		
Mean ± SD (g)	952.5 ± 363.6	521.7 ± 336.2
Mean ± SEM^a^	952.5 ± 71.3	521.7 ± 65.9

a Calculated difference in weight gain of 1‐month‐old infants of normal‐weight and obese mothers: 430.8 ± 97.1 (*P *<* *0.0001 at 95% CI by paired *t*‐test); AUC = 0.82 (positive group − normal‐weight).

The protein expression patterns of all samples (two groups of 26 each, normal weight and obese) were obtained on two types of ProteinChip arrays, CM10 and Q10, and the spectral data were submitted to several statistical analyses: univariate Mann–Whitney *U* test, ROC curve analysis, multivariate heat maps/hierarchical clustering, and PCA.

**Figure 1:**
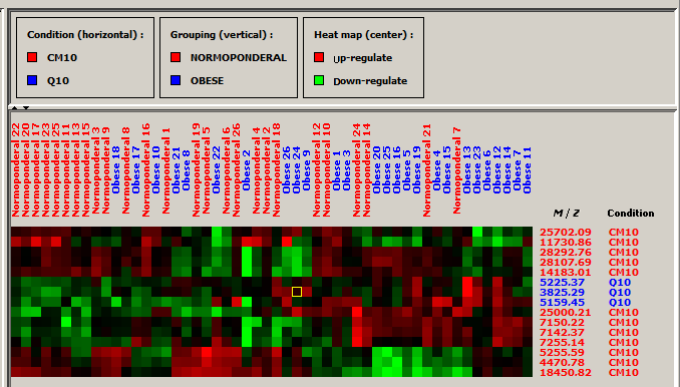
Heat map/hierarchical clustering of 15 breast milk proteins of 52 mothers divided into two groups, normal weight (*n* = 26) and obese (*n* = 26). The clusters are obtained by combining the average intensity values of the samples tested in duplicate on CM10 and Q10 ProteinChip arrays using the following acquisition protocol: laser energy, 3500 nJ; focus mass, 10 000 Da; matrix attenuation, 3000 Da. Above the heat map are shown the sample names (numbered from 1 to 26 in each group, in red for the mothers of normal weight (normoponderal), and in blue for the obese mothers). On the right side of the image are the molecular masses detected on ProteinChip arrays of CM10 (red) and Q10 (blue) type. EDM conditions: first pass: peak S/N, ≥ 5; valley depth S/N, ≥ 2; minimal peak threshold, 20% of all spectra; second pass: peak S/N, ≥ 2; valley depth S/N, ≥ 2; third pass: adding estimated (missing) peaks to complete clusters; clustered mass window width, 0.1%; *m*/*z* range of analysis (*z* = 1), 3000–30 000 Da.

In the Mann–Whitney test, 15 proteins discriminating both groups were found (Table 2; Data S2). Of these, seven were overexpressed in the obese group (3825.3, 5159.4, 5225.4, 7142.4, 7150.2, 7255.1, 25000.2 Da) and eight in the normal‐weight group (4470.8, 5255.6, 11730.9, 14183.0, 18450.8, 25702.1, 28107.7, 28292.8 Da). A heat map/hierarchical clustering of these 15 proteins allowed discrimination to some extent of the two studied groups with two clear‐cut blocks, the first one encompassing 10 members of the normal‐weight group, and the second one comprising seven members of the obese group (Fig. 1).

**Table 2:** Candidate biomarkers discriminating between obese and normal‐weight groups

*m*/*z* a	*P* b	AUC^c^	Array
3825.29 ± 1.37	0.0002	0.75	Q10
5225.37 ± 3.99	0.0004	0.72	Q10
28107.69 ± 11.09	0.0065	0.32	CM10
5159.45 ± 5.33	0.0087	0.67	Q10
7150.22 ± 1.34	0.0171	0.67	CM10
4470.78 ± 1.22	0.0227	0.36	CM10
25702.09 ± 26.43	0.0227	0.37	CM10
28292.76 ± 15.32	0.0267	0.36	CM10
7255.14 ± 1.76	0.0281	0.67	CM10
14183.01 ± 5.41	0.0282	0.31	CM10
7142.37 ± 1.54	0.0290	0.63	CM10
25000.21 ± 29.28	0.0306	0.66	CM10
18450.82 ± 24.89	0.0314	0.35	CM10
11730.86 ± 2.87	0.0368	0.37	CM10
5255.59 ± 1.35	0.0368	0.36	CM10

a The mass‐to‐charge ratios (*m*/*z*) corresponds to molecular masses expressed in Da (*z *=* *1) ± SD.

b Significance of expression difference: 0.0002 < *P *<* *0.04 at 95% CI (Mann–Whitney *U* test).

c In all AUC the obese group is selected as positive.

**Figure 2:**
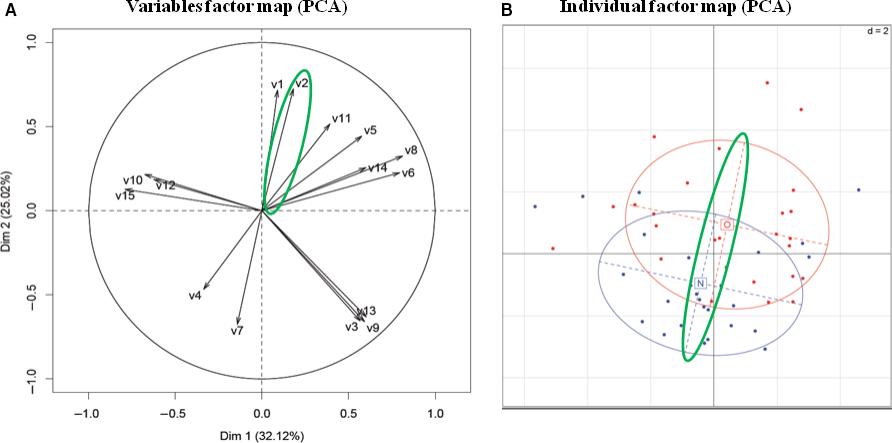
PCA of the 15 significant proteins (Table 2) denoted to as ‘v1–v15’. The tendency of separation is shown by green ellipses. Blue and red dots indicate individual data items of normal weight (N) and obese (O) mothers, respectively. The tendency of separation in two groups by the pIgR (‘v2’; panel A) is in line with the vertical axis between the two centers of gravity of the obese (‘O’) and normal weight (‘N’) groups (panel B). However, this tendency is not definite and can also be attributed to p3825 (‘v1’). Software used for the PCA: r package, version 3.2.2 ( https://CRAN.R-project.org/doc/FAQ/R-FAQ.html; K. Hornik, 2016).

An additional variables factor map (PCA) of the expression differences of these 15 proteins confirmed the same tendency of separation in two groups, with seven proteins for the obese and eight for the normal‐weight mothers. The first and the second PCA axis, corresponded respectively to 25.02% and 32.12% of the variance, revealing a strong positioning of the protein biomarkers for obese mothers in only one quadrant (upper right). Inversely, the positioning of protein biomarkers for normal‐weight mothers extended over the three other quadrants, according to their PCA distribution (Fig. 2A). Furthermore, the individual factor map provided evidence for the two most marked proteins, namely ‘p5225’ (5225.4 Da) and, to a lesser extent, ‘p3825’ (3825.3 Da), both overexpressed in the group of obese mothers (Fig. 2B).

For this reason, p5225 was selected for further purification with a view to identification by sequencing. It was purified by several steps including ion exchange chromatography, reversed phase HPLC and Tricine SDS/PAGE. Throughout purification, all obtained fractions were systematically tested for the presence of the target p5225 protein by SELDI (the ion exchange chromatography fractions) and MALDI (the reversed phase HPLC fractions and those of passive elution from the Tricine SDS/PAGE gel slices). Eventually, the gel slice containing purified p5225 was trypsinized, submitted to LC‐MS/MS, and the determined peptide masses were compared *in silico* to those in the proteome database of *H. sapiens*. In this way a 16‐amino‐acid sequence (ASVDSGSSEEQGGSSR) was identified, which is part of the larger 49‐amino‐acid p5225 found by SELDI, i.e. REIENKAIQDPRLFAEEKAVADTRDQADGSRASVDSGSSEEQGGSSRAL (Data S3). The BLAST analysis undoubtedly shows that p5225 lies within the extracellular region of the polymeric immunoglobulin receptor (pIgR). The main functions of pIgR are to mediate transcellular transport of dimeric IgA across mucosal epithelia and to serve as a precursor of the secretory component (pIgR:CAA51532.1; http://www.uniprot.org/uniprot/P01833).

## Discussion

The innovative point of this study is the establishment of a discriminant breast milk proteomic pattern between dyads of obese mothers whose infants have delayed weight gain and normal‐weight mothers whose infants of the same age have normal weight gain. This pattern consists of 15 candidate biomarkers with both a qualitative (*m*/*z*) and a semi‐quantitative (intensity) feature. In our experiments, we used the delipidized milk fraction (whey) and SELDI methodology, which detects preferentially high abundance proteins. A search for low abundance proteins necessitates their prior enrichment in human milk by specific methods 15, 16, 17 and could possibly complement this pattern.

The identified MS/MS sequence of the p5225 candidate biomarker allows localization of it at position 591–640 of the full‐length pIgR molecule, which corresponds to the sixth domain of pIgR 18, 19. Domain 6 of pIgR is involved in the enzymatic cleavage of pIgR and the release of a free secretory component into the intestinal lumen 19, and this may function as well in the epithelium of the mammary gland. Interestingly, the sequences of 10 amino acids flanking the N‐ and C‐terminal ends of the intermediary highly conserved nine‐amino‐acid sequence at position 605–613 within domain 6 has been shown to exert opposite effects on pIgR cleavage, by enhancing and reducing cleavage efficiency, respectively. Since all breast milk samples in our experiments were supplemented, as soon as collected, with one of the most exhaustive combinations of protease inhibitors available (i.e. ‘Complete, EDTA‐free’; Roche), the origin of the p5225 fragment supposes (a) enzymatic cleavage of the extracellular domain of pIgR on the apical surface of polarized epithelial cells 19, and (b) rapid enzymatic hydrolysis in mature milk that has overridden the protection of the protease inhibitors used. In the latter case, it is likely that full‐length pIgR is very rapidly degraded during the first 20–30 min process of breast milk collection. This is corroborated by a recent study that pinpoints pIgR as one of the two human milk proteins that are most affected by enzymatic hydrolysis 20, albeit some enzymes still remain unidentified 19.

However, the relative abundance of the p5225 fragment detected by SELDI may not only be due to increased protease activity and rate of hydrolysis, but also reflect overexpression of the full‐length pIgR and pertains to our main finding that mature breast milk of obese mothers which are fully breastfeeding their children may have a higher pIgR content than that of matched normal‐weight mothers. The putative involvement of pIgR in the weight control of breastfed infants is corroborated by two independent functional studies on an animal model. In this latter regard transgenic experiments on mice have convincingly shown that the targeted overexpression of *pIgR* in the mammary gland impairs the nutritional value of milk 21, which results in retarded growth and development of the newborn pups (regardless of whether they were transgenic or not) drinking this milk, eventually leading to death 2 weeks after birth 22. These experiments provide a plausible explanation for a novel pIgR function deduced from our exploratory study, i.e. correlation of pIgR overexpression to some delay in the early infants’ weight gain. Moreover, qualitative analysis of transgenic murine milk has revealed that overexpression of *pIgR* caused the loss of κ‐casein and the appearance of serum amyloid A‐1 (an acute inflammatory protein) 22. The two latter studies imply that the loss of the first protein, the overexpression of the second, or a combination of both has caused growth disturbance of the suckling pups, but obviously the underlying pathogenesis is much more complex. It has been shown that the induction of *pIgR* in the mammary gland affects expression of a much larger number of genes and their final protein products in milk, as documented by patterns of intestinal cell gene expression in breeding experimental schemes with *pIgR*‐sufficient and *pIgR*‐deficient mice 23.

Although tantalizing, our hypothesis of pIgR overexpression in obese mothers needs definite demonstration by more sophisticated methods, such as absolute MS quantification using internal standards. The reported proteomic SELDI pattern and the sequencing of the pIgR biomarker open perspectives for further deciphering the complex multifactorial breast milk‐mediated molecular cross‐talk between mother and infant.

## Conflict of interest

The authors declare no conflict of interest.

## Author contributions

CL participated in the collection of breast milk samples within the frame of a clinical trial registered at the EudraCT Clinical Trials Registry (eudract.ema.europa.eu) as 2009‐A00912‐55. EV performed SELDI EDM, MS‐assisted purification of target proteins and statistical analysis. MP contributed to all SELDI and protein purification experiments. RR contributed to the statistical analysis. SC did the LC‐MS/MS sequencing. RH organized and supervised the clinical part of the study (case–control criteria, mothers’ and infants’ parameters, breast milk collection), and participated in writing the manuscript. CA conceived the proteomic part of the study, conducted all SELDI and peptide purification experiments, analyzed the obtained data and wrote the manuscript.

## Supporting information


**Data S1.** Clinical data.

**Data S2.** Spectral data.

**Data S3.** Full‐length pIgR, 16 AA peptide and 49 AA ‘p5225’.
