# Supplementary material for: Proteomic pattern of breast milk discriminates obese mothers with infants of delayed weight gain from normal‐weight mothers with infants of normal weight gain
Source: FEBS Open Bio. 2019 Mar 15;9(4):736–42. doi: 10.1002/2211-5463.12610 (PMC6443869; doi:10.1002/2211-5463.12610)
Supplement: Supplementary file 3 — Data S3. Full‐length pIgR, 16 AA peptide and 49 AA ‘p5225’. [file FEB4-9-736-s003.docx]

**Full-length polymeric immunoglobulin receptor precursor (pIgR), 49 amino acid p5225 and deriving 16 amino acid peptide found by MS/MS**

**Polymeric immunoglobulin receptor precursor [Homo sapiens]**

>NP_002635.2 polymeric immunoglobulin receptor precursor [Homo sapiens]

MLLFVLTCLLAVFPAISTKSPIFGPEEVNSVEGNSVSITCYYPPTSVNRHTRKYWCRQGARGGCITLISSEGYVSSKYAGRANLTNFPENGTFVVNIAQLSQDDSGRYKCGLGINSRGLSFDVSLEVSQGPGLLNDTKVYTVDLGRTVTINCPFKTENAQKRKSLYKQIGLYPVLVIDSSGYVNPNYTGRIRLDIQGTGQLLFSVVINQLRLSDAGQYLCQAGDDSNSNKKNADLQVLKPEPELVYEDLRGSVTFHCALGPEVANVAKFLCRQSSGENCDVVVNTLGKRAPAFEGRILLNPQDKDGSFSVVITGLRKEDAGRYLCGAHSDGQLQEGSPIQAWQLFVNEESTIPRSPTVVKGVAGGSVAVLCPYNRKESKSIKYWCLWEGAQNGRCPLLVDSEGWVKAQYEGRLSLLEEPGNGTFTVILNQLTSRDAGFYWCLTNGDTLWRTTVEIKIIEGEPNLKVPGNVTAVLGETLKVPCHFPCKFSSYEKYWCKWNNTGCQALPSQDEGPSKAFVNCDENSRLVSLTLNLVTRADEGWYWCGVKQGHFYGETAAVYVAVEERKAAGSRDVSLAKADAAPDEKVLDSGFREIENKAIQDPRLFAEEKAVADTRDQADGSRASVDSGSSEEQGGSSRALVSTLVPLGLVLAVGAVAVGVARARHRKNVDRVSIRSYRTDISMSDFENSREFGANDNMGASSITQETSLGGKEEFVATTESTTETKEPKKAKRSSKEEAEMAYKDFLLQSSTVAAEAQDGPQEA

*Text in red : The 16 amino acid sequence ASVDSGSSEEQGGSSR determined by LC-MS/MS (its original spectrum is given below).*

*Highlighted in yellow : This 16 amino acid sequence ASVDSGSSEEQGGSSR is part of the larger 49 amino acid fragment fragment “p5225” found by SELDI i.e. : REIENKAIQDPRLFAEEKAVADTRDQADGSRASVDSGSSEEQGGSSRAL*


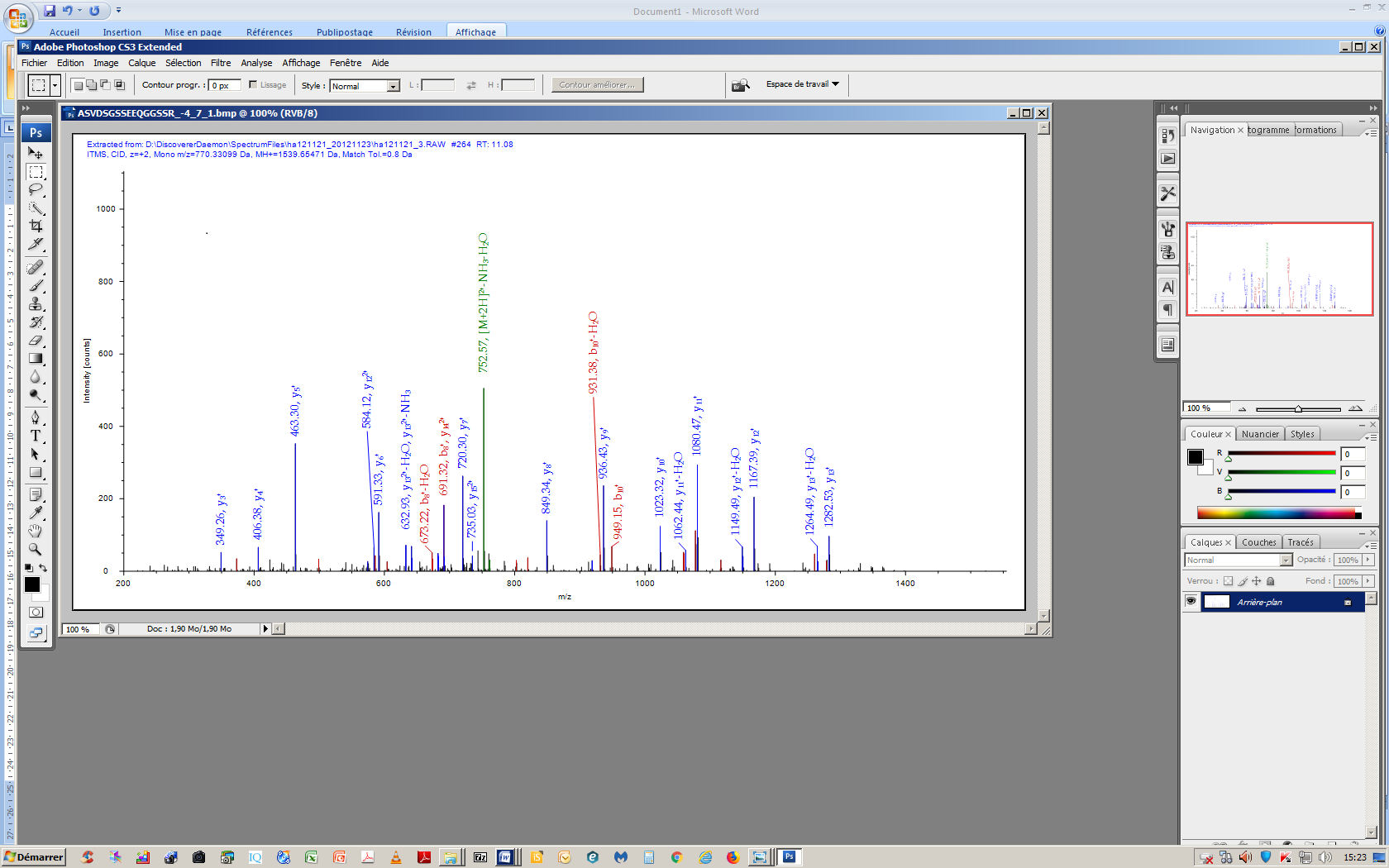


Original MS/MS spectrum of the 16 amino acid sequence ASVDSGSSEEQGGSSR.

**Trypsin cleavage sites of the 49 amino acid p5225**


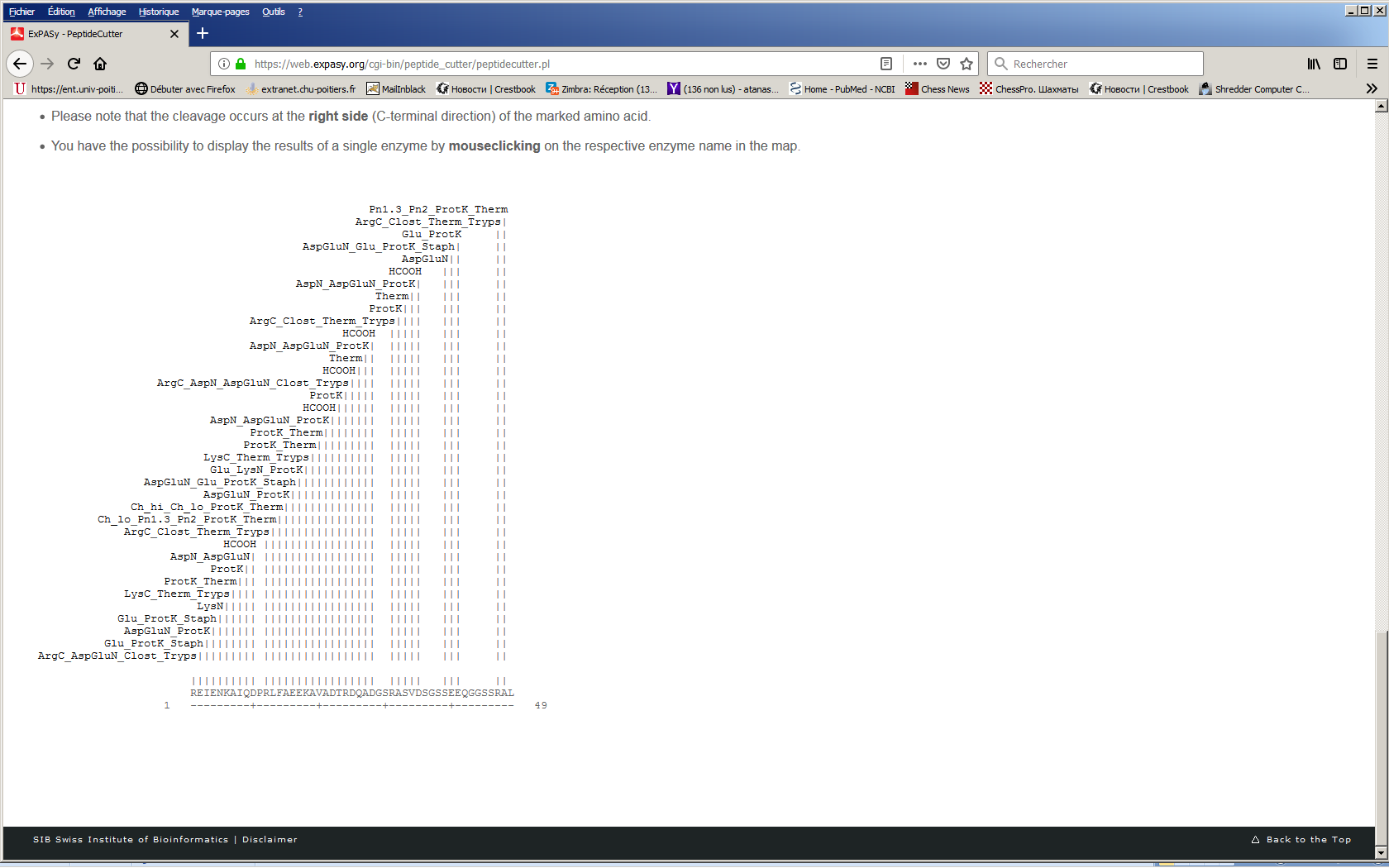


| **Name of enzyme** | | **No. of cleavages** | | **Positions of cleavage sites** | |
| --- | --- | --- | --- | --- | --- |
| [Trypsin](https://web.expasy.org/peptide_cutter/peptidecutter_enzymes.html#Tryps) | 7 | | 1 6 12 18 24 31 47 | |  |
|  |  | |  | |  |

**R**EIEN**K**AIQDP**R**LFAEE**K**AVADT**R**DQADGS**R**ASVDSGSSEEQGGSS**R**AL

<https://web.expasy.org/peptide_cutter/>
